# Associations between urea nitrogen and risk of depression among subjects with and without type 2 diabetes: A nationwide population-based study

**DOI:** 10.3389/fendo.2022.985167

**Published:** 2022-10-27

**Authors:** Yafei Mao, Xinyuan Li, Shumin Zhu, Jin Ma, Yulan Geng, Yuanyuan Zhao

**Affiliations:** ^1^ Department of Laboratory Medicine, The First Hospital of Hebei Medical University, Shijiazhuang, China; ^2^ Department of Laboratory Medicine, The Third Hospital of Hebei Medical University, Shijiazhuang, China

**Keywords:** blood urea nitrogen, diabetes, T2D, depression, national health and nutrition examination

## Abstract

**Background:**

Depression and type 2 diabetes (T2D) are serious public health problems with irreversible health consequences and a significant economic burden on the healthcare system. Previous studies have suggested that blood urea nitrogen (BUN) was inversely longitudinally associated with incidence of diabetes and depression in adults, but few well-designed studies have examined the effects of status of T2D on the full range of relationship between BUN and depression.

**Methods:**

The analysis sample consisted of adults aged≥20 years from the 2007-2014 National Health and Nutrition Examination Survey (NHANES) who completed the Patient Health Questionnaire-9 (PHQ–9), involving 19,005 participants. By stratifying participants according to T2D status, we further assessed the difference between BUN and risk of depression in participants with and without T2D using multivariate logistic regression (interaction test).

**Results:**

In this cross-sectional study, the association between BUN and depression prevalence appeared to differ between the T2D and non-T2D groups (OR: 1.00, 95% Cl: 0.95-1.05 vs. OR: 0.89, 95% Cl: 0.85-0.93). In addition, there was evidence of an interaction between BUN levels and T2D status in reducing the risk of depression (*P* value for interaction = 0.032.) The relationship between BUN and depressive symptoms was significant in non-T2D subjects (*P* < 0.001), but not in T2D (*P* = 0.940).

**Conclusions:**

Our findings suggest that there is a significant relationship between BUN and depression, and T2D status may influence the association between BUN and the risk of depression. Such findings require further prospective studies to provide more evidence.

## Introduction

Depression, one of the most common and distressing disorders worldwide, is characterized by a very depressed mood in all aspects of life and an inability to experience a sense of happiness (1).The co-occurrence of diabetes and depression has increasingly become a global challenge (2). Epidemiological research studies (3, 4) have shown that people with chronic conditions, such as diabetes, are twice as likely to develop mental health comorbidities (such as depression and anxiety) (5, 6) as the general healthy population, adversely affecting quality of life and diabetes outcomes (7–10). The social isolation, economic stress, and other effects of the COVID-19 pandemic exacerbate the burden of depression (11), and major depression increases the risk of other illnesses and suicide. An increasing number of studies have shown that microvascular dysfunction is a common phenomenon in patients with diabetes, including effects on the brain and nerves, and that diabetes-related microvascular dysfunction is associated with a high risk of depression, stroke, and cognitive dysfunction (12). In patients with diabetes mellitus with depression, they may possess a poorer self-management ability, a poorer degree of glycemic control and a higher mortality (13, 14). The mechanisms underlying the development of concomitant depression in patients with diabetes remain poorly understood. There is evidence of their bi-directional association, with possible shared biological determinants (15).

Urea, the end product of protein metabolism, plays an important role in reducing insulin sensitivity (16) Also, urea contributes to depression by damaging the medial prefrontal cortex (17). A study conducted by Li et al. found that mice with ineffective urea transporter B (UT-B), resulting in the accumulation of urea in the blood and brain, exhibited depression-like behavior (18). Furthermore, Wang et al. verified that high levels of urea contribute to depression by mimicking mice with chronic kidney disease (CKD) in humans, and additionally, in human trials with patients in the uremic phase of chronic renal failure came to similar conclusions, and their findings suggest that high urea in the brain induces depression under physiological and pathological conditions, independent of stress (17). Boer et al. illustrated that patients with chronic kidney disease (CKD) have lower insulin sensitivity, decreased insulin clearance, and inadequate enhancement of insulin secretion (19, 20). Reviews by Thomas et al. (21) and Koppe et al. (22)suggest that defects in insulin secretion in patients with CKD are caused by elevated levels of circulating urea, which become evident especially in the advanced stages of CKD. In a mouse model simulating CKD, experiments confirmed the above, indicating that chronic accumulation of urea causes β-cell dysfunction in CKD patients and that urea is responsible for reduced insulin sensitivity and defective insulin secretion (22, 23). However, in several other studies, BUN and depression risk were negatively correlated, demonstrated in the Korean and Chinese populations respectively (24, 25).

Numerous studies have shown higher BUN to be associated with an increased risk of developing diabetes mellitus (16, 26, 27). However, these studies did not focus on depressed patients with concomitant diabetes. More importantly, there are no population-based studies examining the relationship between BUN and the risk of depression. Therefore, this study hypothesized that there is also a correlation between urea levels and risk of depression in the general population and that this may be influenced by T2D status.

Herein, we aimed to compare the relationship between BUN and risk of depression between a large sample of adults with and without T2D in the general population. To increase the reliability of the analysis, we used four independent waves of data from the National Health and Nutrition Examination Survey (NHANES).

## Materials and methods

### Data sources and study population

The National Health and Nutrition Examination Survey (NHANES) is an ongoing series of sample surveys in which participants are selected using a multistage, stratified probability approach (28), designed to collect nationally representative data from the noninstitutionalized U.S. population. An extensive household interview was conducted to collect demographic and health history data. A Mobile Examination Center (MEC) was used for conducting physical examinations and collecting blood samples. Serum samples were examined at the Laboratory Sciences Division of the National Center for Environmental Health at the Centers for Disease Control and Prevention.

The study was approved by the National Center for Health Statistics Research Ethics Review Board. Our study was a cross-sectional research project, based on publicly available data from NHANES (2007- 2014), with all details from the official website (https://www.cdc.gov/nchs/nhanes/about_nhanes.htm). The original study protocol is available on the NHANES Ethics Review Committee website (https://www.cdc.gov/nchs/nhanes/irba98.htm), with formal approval of the Ethics Review Committee (protocol #2005-06; #2011-17). Participants provided written informed consent at the time of registration for the study. Participants in our study were over 20 years and had completed interviews and assessments at a MEC. Participants were excluded if they had missing data on depressive status, T2D status and covariates.

### Depressive symptoms

The Patient Health Questionnaire (PHQ–9), a nine-item screening tool that measures the frequency of a variety of depressive symptoms within the previous two weeks (29), was used to assess depression status in NHANES. Each of the 9 items consists of responses on a four-point scale, with 0 = “not at all,” 1 = “a few days,” 2 = “more than half the days,” and 3 = “almost every day,” for a total score of 0 to 27. A score of ≥10 was used as a cut point for participation in the depression group. For major depression, it had an 88% sensitivity and 88% specificity (30) and suggested a moderate to severe level of depressive symptoms.

### Blood urea nitrogen

Blood samples collected for BUN measurement during the MEC examination are processed and stored in appropriate frozen (-30°C) conditions until shipped to National Center for Environmental Health for testing. Detailed specimen collection and handling instructions are discussed in the NHANES Laboratory/Medical Technician Procedures Manual (LPM). In this study, BUN was classified into three categorical variables.

### Identification of T2D

The American Diabetes Association criteria (31) and a self-report questionnaire were used to define T2D cases. T2D patients were defined as those with fasting blood glucose (FPG)≥126 mg/dL, glycated hemoglobin (HbA1c)≥6.5 percent, 2-h plasma glucose≥200 mg/dL during an oral glucose tolerance test (OGTT), self-report questionnaire data suggesting physician diagnosis of diabetes, and current usage of insulin or diabetic pill to decrease blood glucose.

### Other covariates

The present study considered the age, gender, race/ethnicity, body mass index (BMI), educational level, smoking status, drinking status, physical activity and laboratory data including white blood cell (WBC), albumin, aspartate aminotransferase (AST), alanine amino transferase (ALT), creatinine, uric acid, lactate dehydrogenase (LDH). Race was classified into five categories (32), including non-Hispanic white, non-Hispanic black, Mexican American, other Hispanic or other. Education level was categorized as less than high school, high school, equivalent and college or above. According to standardized protocols, BMI was calculated as weight in kilograms divided by height in meters squared and was categorized as <25.0, 25.0 to <30.0, and ≥30.0 kg/m^2^. Smoking status was categorized as smokers and never smokers. Smokers were defined as participants who had smoked more than 100 cigarettes in the past; conversely those who had not even smoked 100 cigarettes in their lifetime were considered never smokers. A respondent was considered as a drinker if they had drunk at least 12 alcohol drinks a year in their life (33). Walking, moderate, and vigorous activities were used to categorize physical activity into three levels of intensity. Self-reported current usage of antihypertensive medications or a doctor’s diagnosis were used to define hypertension.

### Statistical analysis

Statistical testing was two-sided with a level of significance set at P = 0.05. All analyses were performed with the Free Statistics software version 1.6 (34) and the statistical software packages R (http://www.R-project.org, The R Foundation). Means ± standard deviations, and frequencies (percentages) were used to express demographic and clinical indicators. The t-test (normal distribution) and Kruskal-Wallis (skewed distribution) tests were used to examine continuous variables. To further analyze the relationship between different doses of blood urea nitrogen and depression, we used univariate and multivariate binary logistic regression. In multivariate logistic regression, we showed (1) unadjusted models, (2) model 1 adjusted covariates including age and gender, and (3) model 2 adjusted for variables from model 1 plus BMI, race and other indicators (p<0.001) including educational level, smoking status, alcohol consumption, albumin, ALT, AST, creatinine, LDH, Uric acid, hypertension, diabetes and physical activities. Participants’ blood urea nitrogen levels and risk of depression were compared among subjects with and without diabetes. The multivariate logistic regression model was used to perform subgroup analysis based on diabetes status. Outliers with blood urea nitrogen levels beyond the range of mean 3 SD (0-10.655 mmol/L) or mean 2 SD (0.030-8.530 mmol/L) were excluded from sensitivity analyses. Interactions between subgroups were examined by likelihood ratio testing.

## Results

### Baseline characteristics of the study population

**Figure 1:**
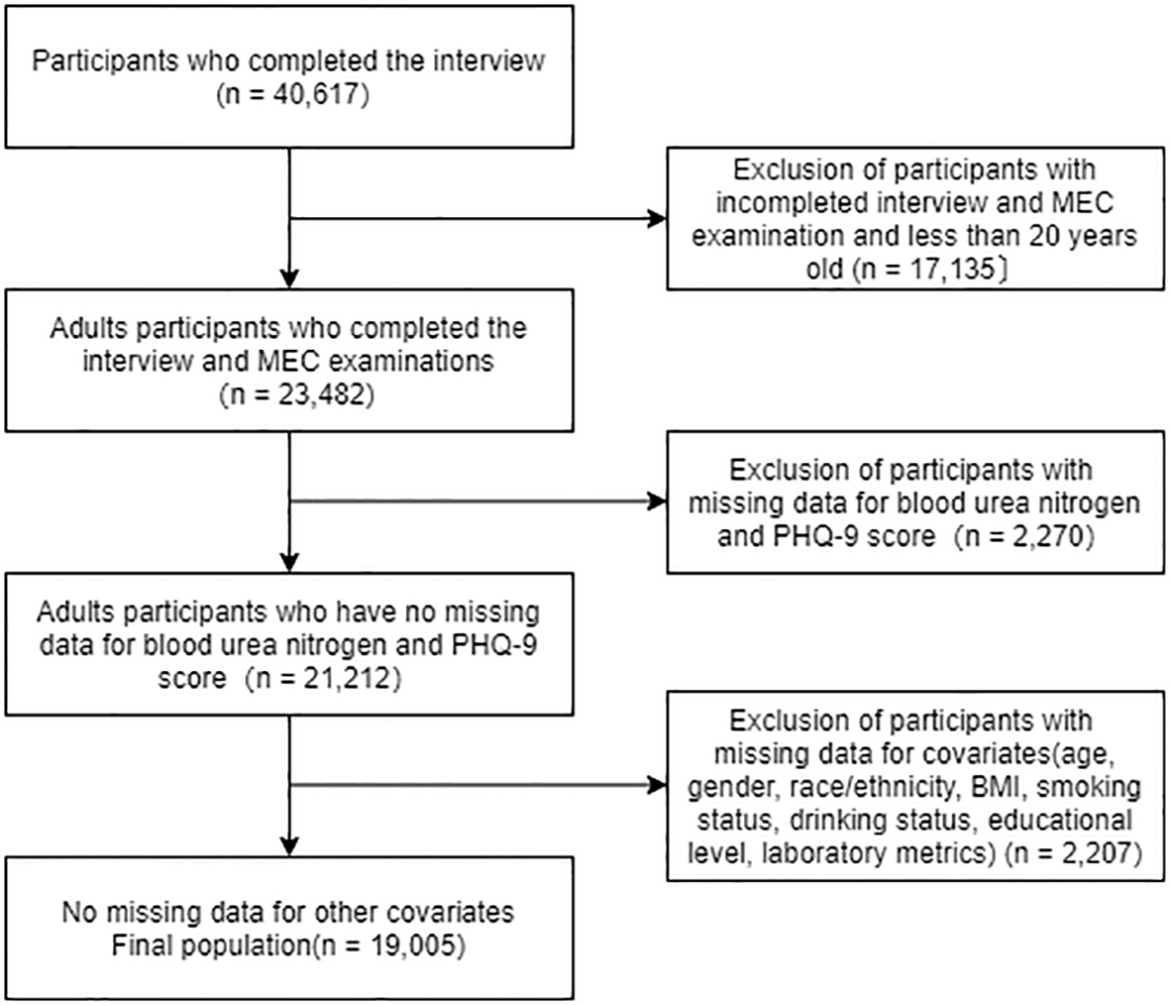
The flow chart of the study.

We analyzed 2007-2014 NHANES data and identified 40,617 potential participants; 23,482 adults (≥20 years) who completed the interview and received the MEC screening. Participants with missing data on blood urea nitrogen and PHQ-9 scores were excluded (n = 2,270). After excluding participants with missing data on covariates (n = 2,207), the remaining 19,005 participants were included in our analysis. A flow chart of the exclusion criteria is shown in **Figure 1**.

Of the 19,005 participants included in the study, 3,422 (18.0%) were diagnosed with diabetes mellitus. Baseline demographic and clinical characteristics compared between patients who were diagnosed with diabetes and those without diabetes are listed in **Table 1**. The diabetes status were confirmed based on the American Diabetes Association criteria (31). Compared with the non-diabetic group, those with diabetes were more likely to be men, older, smoker, less-educated, and mostly had a higher BMI. Additionally, the group of diabetes had more high-grade disease such as hypertension and depression. Individuals with diabetes had lower albumin and a lower work activity and recreational activity level. For laboratory metrics, WBC, ALT, AST, BUN, creatinine, LDH, uric acid was lower in participants who without diabetes (all *P <*0.001).

**Table 1:** Baseline characteristics of participants.

Covariates	Total	Without T2D	With T2D	*P*-value
	(n = 19005)	(n = 15583)	(n = 3422)
**Age (years)**	49.5 ± 17.7	47.0 ± 17.5	61.2 ± 13.4	<0.001
**Gender, n (%)**				0.003
Female	9598 (50.5)	7948 (51)	1650 (48.2)	
Male	9407 (49.5)	7635 (49)	1772 (51.8)	
**Education level, n (%)**				<0.001
Did not graduate from high school	4804 (25.3)	3562 (22.9)	1242 (36.3)	
Graduated from high school	4335 (22.8)	3533 (22.7)	802 (23.4)	
College education or above	9866 (51.9)	8488 (54.5)	1378 (40.3)	
**Race/Ethnicity, n (%)**				<0.001
Mexican American	2849 (15.0)	2264 (14.5)	585 (17.1)	
Other Hispanic	1935 (10.2)	1550 (9.9)	385 (11.3)	
Non-Hispanic white	8663 (45.6)	7332 (47.1)	1331 (38.9)	
Non-Hispanic black	3808 (20.0)	2958 (19)	850 (24.8)	
Other races	1750 (9.2)	1479 (9.5)	271 (7.9)	
**BMI (kg/m^2^), Mean ± SD**	29.1 ± 6.8	28.4 ± 6.5	32.3 ± 7.4	<0.001
**BMI, n (%)**				<0.001
< 25 kg/m^2^	5521 (29.1)	5062 (32.5)	459 (13.4)	
25 to < 30 kg/m^2^	6379 (33.6)	5372 (34.5)	1007 (29.4)	
≥ 30 kg/m^2^	7105 (37.4)	5149 (33)	1956 (57.2)	
**Laboratory Metrics**				
WBC, (×10^9^/L)	7.2 ± 2.4	7.1 ± 2.5	7.5 ± 2.2	<0.001
Albumin, (g/L)	42.4 ± 3.4	42.7 ± 3.3	41.4 ± 3.4	<0.001
ALT, (U/L)	25.5 ± 21.6	25.1 ± 18.4	27.6 ± 32.4	<0.001
AST, (U/L)	26.0 ± 17.1	25.7 ± 16.5	27.4 ± 19.4	<0.001
BUN, (mmol/L)	4.8 ± 2.1	4.5 ± 1.8	5.7 ± 3.0	<0.001
Creatinine (µmol/L)	80.2 ± 40.6	90.4 ± 65.1	< 0.001
LDH, (U/L)	130.4 ± 32.4	129.4 ± 31.3	135.3 ± 36.6	<0.001
Uric acid (µmol/L)	326.2 ± 85.9	321.4 ± 83.4	347.9 ± 93.4	<0.001
**Smoking status, n (%)**				<0.001
≥100 cigarettes during their lifetime	8647 (45.5)	6918 (44.4)	1729 (50.5)	
**Drinking status, n (%)**				<0.001
≥ 12 alcohol drinks a year	13794 (72.6)	11606 (74.5)	2188 (63.9)	
**Physical activity, n (%)**				
Vigorous work activity	3516 (18.5)	3091 (19.8)	425 (12.4)	<0.001
Moderate work activity	6755 (35.5)	5784 (37.1)	971 (28.4)	<0.001
Walk or bicycle	4996 (26.3)	4319 (27.7)	677 (19.8)	<0.001
Vigorous recreational activities	3981 (20.9)	3742 (24)	239 (7)	<0.001
Moderate recreational activities	7639 (40.2)	6586 (42.3)	1053 (30.8)	<0.001
**Hypertension, n (%)**	6871 (36.2)	4629 (29.7)	2242 (65.5)	<0.001
**Depression, n (%)**	1784 (9.4)	1325 (8.5)	459 (13.4)	<0.001

BMI, body mass index; T2D, type 2 diabetes; WBC, white blood cell; ALT, alanine aminotransferase; AST, aspartate aminotransferase; BUN, blood urea nitrogen; LDH, Lactate dehydrogenase.

### Blood urea nitrogen affects the risk of depression patients

The univariate analysis in **Table 2** indicated that gender, education level, BMI, smoking status, physical activity, WBC, albumin, AST, BUN, LDH, uric acid and hypertension were associated with depression. **Table 3** shows the results of logistic regression analyses. In the non-adjusted model, blood urea nitrogen was inversely associated with the risk of depression (OR, 0.95; 95% CI, 0.92–0.97). Results were similar after adjusting for age and gender (OR, 0.97; 95% CI, 0.95–1.00). Multivariate regression models were performed to adjust for other possible confounders, including BMI, race, educational level, smoking status, alcohol consumption, albumin, ALT, AST, creatinine, LDH, Uric acid, hypertension, diabetes and physical activities, the inverse relationship remained significant (P < 0.001). The significant relationship also exists in different adjustment models when blood urea nitrogen was transformed into a categorical variable (P < 0.001).

**Table 2:** Association of covariates and depression risk.

Variable	OR_95 CI%	*P*-value
**Age (years)**	1.00 (0.99~1.00)	0.053
**Gender, n (%)**		
Female	1 (reference)	
Male	0.51 (0.46~0.57)	<0.001
**Education level, n (%)**		
Did not graduate from high school	1 (reference)	
Graduated from high school	0.65 (0.57~0.74)	<0.001
College education or above	0.46 (0.41~0.51)	<0.001
**Race/Ethnicity, n (%)**		
Mexican American	1 (reference)	
Other Hispanic	1.45 (1.21~1.75)	<0.001
Non-Hispanic white	0.96 (0.83~1.11)	0.554
Non-Hispanic black	1.05 (0.89~1.24)	0.538
Other races	0.65 (0.52~0.82)	<0.001
**Laboratory Results**		
WBC, (×10^9^/L)	1.07 (1.05~1.09)	<0.001
Albumin, (g/L)	0.93 (0.91~0.94)	<0.001
ALT, (U/L)	1.00 (1.00~1.00)	0.072
AST, (U/L)	1.00 (1.00~1.00)	0.024
BUN, (mmol/L)	0.95 (0.92~0.97)	<0.001
Creatinine (µmol/L)	1.00 (1.00~1.00)	0.434
LDH, (U/L)	1.001 (1~1.003)	0.028
Uric acid (µmol/L)	1.00 (1.00~1.00)	0.001
**BMI, n (%)**	1.04 (1.03~1.05)	<0.001
**Smoking status, n (%)**		
≥100 cigarettes during their lifetime	1.86 (1.68~2.05)	<0.001
**Drinking status, n (%)**		
≥ 12 alcohol drinks a year (%)	0.95 (0.85~1.06)	0.348
**Physical activity, n (%)**		
Vigorous work activity	0.92 (0.81~1.05)	0.223
Moderate work activity	0.78 (0.71~0.87)	<0.001
Walk or bicycle	0.82 (0.73~0.92)	0.001
Vigorous recreational activities	0.38 (0.32~0.45)	<0.001
Moderate recreational activities	0.44 (0.39~0.49)	<0.001
**Hypertension, n (%)**	1.68 (1.53~1.86)	<0.001

Data presented are ORs and 95%Cls.

BMI, body mass index; WBC, white blood cell; ALT, alanine aminotransferase; AST, aspartate aminotransferase; BUN, blood urea nitrogen; LDH, Lactate dehydrogenase.

**Table 3:** Weighted odds ratios (95% confidence intervals) of depression and different blood urea nitrogen in different models.

Blood urea nitrogen, (mmol/L)	Cases/participants	Non-adjusted Model	Model 1	Model 2
Blood urea nitrogen	1784/19005	0.95 (0.92~0.97)	0.97 (0.95~1.00)	0.94 (0.91~0.97)
*P*-value		<0.001	0.061	<0.001
Subgroups				
Quartile 1	742/6294	1.00(Ref.)	1.00(Ref.)	1.00(Ref.)
Quartile 2	442/5201	0.69 (0.61~0.79)	0.75 (0.66~0.85)	0.83 (0.73~0.95)
Quartile 3	600/7510	0.65 (0.58~0.73)	0.72 (0.64~0.82)	0.75 (0.66~0.86)
*P*-trend		<0.001	<0.001	<0.001

Model 1 was adjusted for age and gender.

Model 2 was additionally adjusted for BMI, race, educational level, smoking status, alcohol consumption, albumin, ALT, AST, creatinine, LDH, Uric acid, hypertension and physical activities.

### Associations between urea nitrogen and risk of depression among subjects with or without T2D

**Figure 2:**
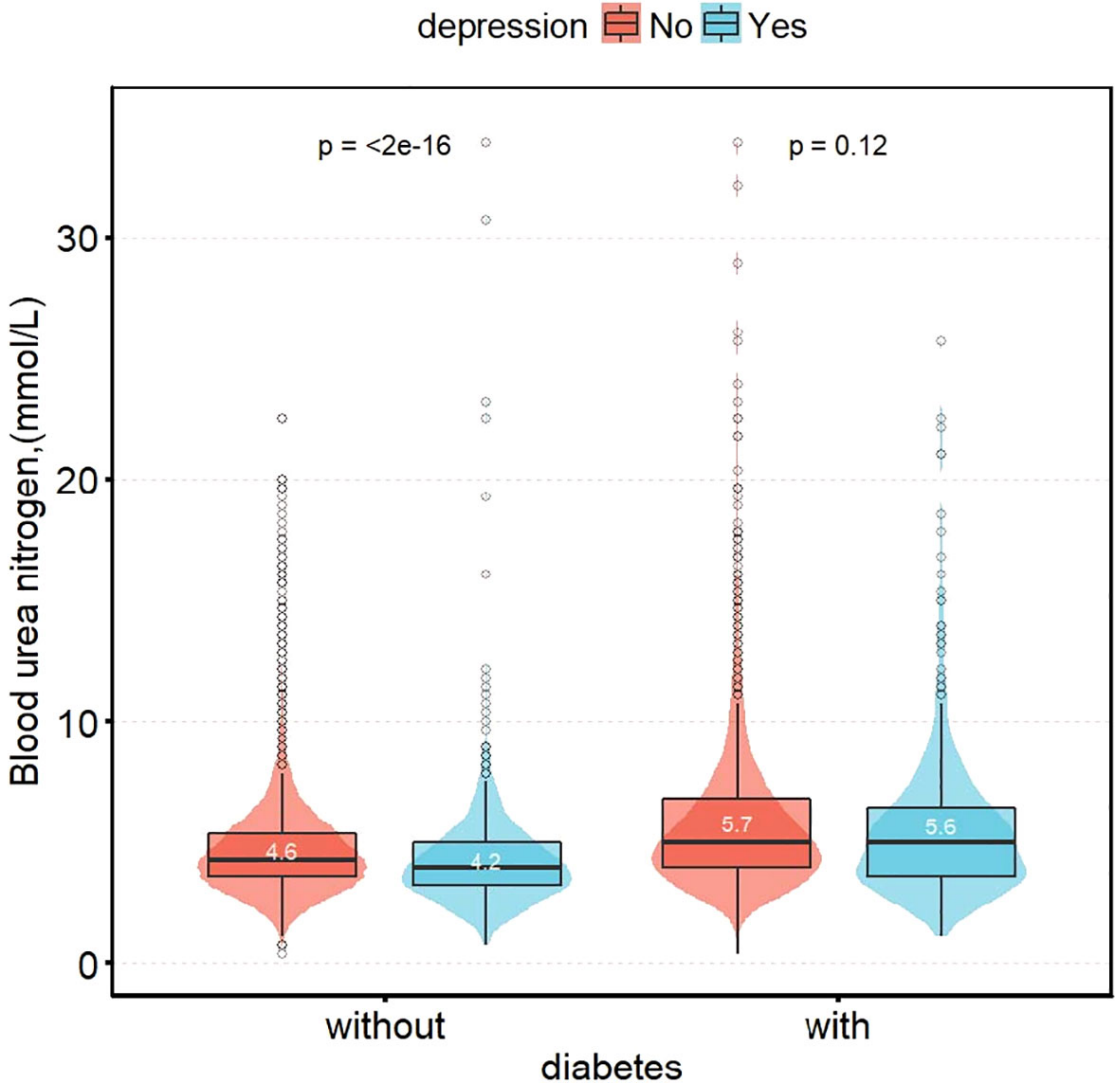
Distribution of blood urea nitrogen in patients with depression group by status of diabetes.

**Figure 2** shows the difference in blood urea nitrogen between non-depression and depression participants. It is not significant in diabetic group (5.7 vs. 5.6 mmol/L, *P* = 0.120). However, in the non-diabetic group, blood urea nitrogen in participants with depression was significantly decreased, compared with non-depression participants (4.6 vs. 4.2 mmol/L, *P* < 0.001).

After adjusting for age, gender, race, BMI, educational level, smoking status, alcohol consumption, albumin, ALT, AST, creatinine, LDH, Uric acid, hypertension and physical activities, as blood urea nitrogen increased, the risk of depression was significantly reduced in the group without T2D (OR: 0.89, 95% Cl: 0.85-0.93; *P* < 0.001), meanwhile, it was not significant in the T2D group (OR: 1.00, 95% Cl: 0.95-1.05; *P* = 0.940). The interaction between T2D status on BUN and depression prevalence was significant (the *P*-value for interaction likelihood ratio test was *P* = 0.032). The interaction remained significant when BUN was transformed into a categorical variable (the *P*-value for the interaction was 0.031) (**Table 4**).

**Table 4:** Interactive effect of blood urea nitrogen and depression in patients with and without T2D (All participants).

Variable	Without T2D (n = 15583)		With T2D (n = 3422)		*P* for interaction
	OR 95% CI	*P*-value	OR 95% CI	*P*-value	
Blood urea nitrogen, (mmol/L)	0.89 (0.85~0.93)	<0.001	1.00 (0.95~1.05)	0.940	0.032
Subgroups					
Quartile 1	1.00 (Ref)		1.00 (Ref)		0.031
Quartile 2	0.85 (0.74~0.99)	0.033	0.74 (0.54~1.01)	0.057	
Quartile 3	0.67 (0.57~0.79)	<0.001	0.99 (0.75~1.32)	0.953	
Trend test		<0.001		0.895	

Adjusted for age, gender, BMI, race, educational level, smoking status, alcohol consumption, albumin, ALT, AST, creatinine, LDH, Uric acid, hypertension and physical activities.

### Threshold effect analysis and sensitive analysis

**Figure 3:**
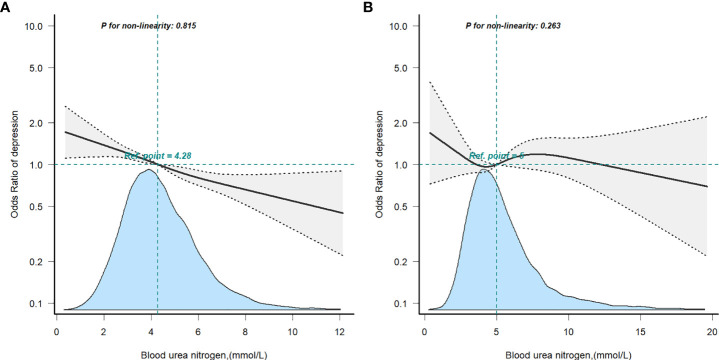
Restricted cubic spline model of the odds ratios of blood urea nitrogen with depression in non-T2D **(A)** or T2D **(B)**. Adjusted for age, gender, race, BMI, educational level, smoking status, alcohol consumption, laboratory Results, physical activity and hypertension.

Restricted cubic spline analysis was applied to explore the dose-response relationship between BUN and the risk of depression in the non-T2D (A) or T2D (B) groups (**Figure 3**), both showing a linear correlation. To back up our conclusions, we conducted sensitivity analyses. After removing subjects with BUN >10.655 mmol/L (mean ± 3SD), the results were stable (**Supplementary Table S1**). The association of BUN with the risk of depression appeared to differ between the T2D group and the non-T2D group (OR: 0.99, 95%Cl: 0.92–1.07 vs. OR: 0.88, 95%Cl: 0.84–0.92) (the *P*-value for the interaction was 0.019). The interaction of BUN on the prevalence of depression in the non-T2D was significant when translated into a categorical variable (the *P*-value for the interaction 0.028). When subjects with BUN >8.530 mmol/L (mean ± 2SD) were excluded (**Supplementary Table S2**), the risk of depression remained different in the T2D and non-T2D groups with increasing BUN (OR: 1.00, 95%Cl: 0.92–1.09 vs. OR: 0.88, 95%Cl: 0.83–0.92) (the *P*-value for the interaction was 0.017). Similarly, when BUN was transformed into a categorical variable, the *P*-value for the interaction was 0.024.

## Discussion

Based on a cross-sectional analysis of US adults (≥20 years) using NHANES (2007-2014) data, we found that serum urea nitrogen was negatively associated with risk of depression when adjusted for potentially important confounders, which is consistent with the results of several previous studies (24, 25), including the Korean (KNHANES) study (24) which showed that BUN and risk of depression were negatively associated in men (OR, 0.87; 95% CI 0.79-0.96) and women (OR, 0.93; 95% CI, 0.88-0.98), and a study from China (n=228) (25) showed that BUN was a protective factor for major depressive disorder. Our findings showed a similar significant association in the depressed population without T2D, whereas the association was not significant in the T2D population.

Urea, an end product of protein metabolism, contributes to reduced insulin sensitivity (16), and also plays an important role in urine concentration processes and water conservation in the mammalian kidney (17). It has long been thought to be of negligible toxicity. Elevated blood urea in patients with chronic renal failure was thought to have no impact on patient survival (35). However, the idea that urea is simply an innocent bystander has recently been challenged by several new observations. Higher urea may have an important role in accelerating atherosclerosis in chronic dialysis patients (36) and in causing depression by damaging the medial prefrontal cortex (17). Wang et al (17) first demonstrated the neurotoxicity of high urea concentrations in a well-documented experiment, and their previous work found that urea transporter protein B (UT-B) deficient mice, in which urea accumulated in blood and brain, exhibited depressive-like behaviour (18). However, UT-As null mice with normal blood urea concentrations showed no depressive-like behaviour (37). The results of the above studies are inconsistent and do not reflect a correlation between BUN and the risk of depression in the general population. Therefore, we examined the association between blood urea nitrogen and risk of depression in the general US adult population (≥20 years) based on NHANES (2007-2014) data and found a negative association, even after adjusting for potentially important confounders.

Findings from many cross-sectional and longitudinal population studies have shown that blood urea nitrogen is associated with an increased risk of diabetes (16, 26, 38), which is consistent with the higher urea levels in the diabetic group of our study population. Their study demonstrated a gradual increase in the risk of diabetes with increasing BUN levels in a cohort of US veterans (16), a cohort of pregnant people (26) and Chinese patients with primary aldosteronism (PA) (38), respectively. However, very few studies have focused on relationship between blood urea nitrogen and depression patients combined with diabetes. In this study, we found that depression risk decreases with higher blood urea levels within a range, and blood urea nitrogen was a protective factor for depression without diabetes (*P* < 0.05). This finding might not apply to depression patients with T2D. Firstly, our previous study (39) showed a negative association between dietary fiber and the risk of depression in the non-T2D population but not in the T2D population. There may be a mechanistic association between T2D patients and depression, leading to biological differences that need to be further investigated. Secondly, microcirculation in the brain is altered in diabetic patients, including increased blood-brain permeability and altered blood flow regulation, and their microvascular dysfunction may cause alterations in metabolic mechanisms when triggering depression (12). Third, this may be related to the relatively small number of people in the T2D group in our study population, which needs to be confirmed in further studies in the future. To our knowledge, this is a systematic and complete study examining the relationship between blood urea nitrogen and the risk of depression among subjects with and without diabetes in a US adult population.

In this study, the associations between BUN and depression in patients with or without T2D were examined after adjusting for variables that could be taken into account. There are several strengths of this study. First, our study population is a large, nationally representative sample of US adults. Second, the study modeled the association between BUN level and depression while adding known and potential covariates for depressive symptoms. Third, this study examined associations stratified by with or without T2D. Fourth, we performed a dose-response analysis to assess the association between BUN and depression quantitatively.

However, there are obvious limitations to our study. First, a cross-sectional study does not allow for causal inference. Second, we cannot rule out the possibility that the observed associations were caused by unmeasured confounders, although we adjusted for various confounding variables. Third, the study covariates were based on self-reporting, and therefore, misinterpretation of the questions or recall issues may arise. Fourth, relatively few of our study population were in the T2D group (n=3422) and the results need to be confirmed in further studies in the future. Fifth, BUN is a single measurement, and it is possible that tests of laboratory indicators, etc., may be influenced by status, such as diet. Nevertheless, the probability of this occurring is very small because NHANES includes an exceptionally large population each year, selected using a multi-stage, stratified probability design. However, despite these limitations, our analyses have previously unachieved statistical power, which provides strong assurance that our results are valid. Nevertheless, replication of the same research questions in other longitudinal data sets with a similar wealth of data or prospective studies is needed.

## Conclusions

In conclusion, these findings demonstrate a significant association between depression and blood urea nitrogen, however this relationship may be confounded by T2D status. Blood urea nitrogen is significantly associated with risk of depression in the non-T2D U.S. population but not in T2D group. This may help to identify new strategies for the prevention and treatment of depression, targeting the general non-T2D population. Although we have provided some clinical clues, further prospective studies are needed to provide more evidence.

## Data availability statement

The original contributions presented in the study are included in the article/**Supplementary Material**. Further inquiries can be directed to the corresponding authors.

## Ethics statement

The studies involving human participants were reviewed and approved by National Center for Health Statistics Research Ethics Review Board. The patients/participants provided their written informed consent to participate in this study.

## Author contributions

YM designed, analyzed, and wrote the manuscript, XL conducted data collection, SZ and JM conducted data interpretation, YG and YZ reviewed the manuscript. All authors contributed to the article and approved the submitted version.

## Funding

This study was supported by Natural Science Foundation of Hebei Province (No. H202120603), Key Research and Development Projects of Hebei Province (No. 18277727D and No. 21377794D).

## Acknowledgments

We thank Dr. Liu jie (People’s Liberation Army of China General Hospital, Beijing, China) for helping in this revision.

## Conflict of interest

The authors declare that the research was conducted in the absence of any commercial or financial relationships that could be construed as a potential conflict of interest.

## Publisher’s note

All claims expressed in this article are solely those of the authors and do not necessarily represent those of their affiliated organizations, or those of the publisher, the editors and the reviewers. Any product that may be evaluated in this article, or claim that may be made by its manufacturer, is not guaranteed or endorsed by the publisher.
