# Supplementary material for: Associations between urea nitrogen and risk of depression among subjects with and without type 2 diabetes: A nationwide population-based study
Source: Front Endocrinol (Lausanne). 2022 Oct 27;13:985167. doi: 10.3389/fendo.2022.985167 (PMC9646599; doi:10.3389/fendo.2022.985167)
Supplement: Supplementary file 1 [file Table_1.docx]

**Supplementary Table S1.** Interactive effect of blood urea nitrogen and depression in patients with and without T2D (All participants). Blood urea nitrogen exclude（X±3SD）

| **Variable** | **Without T2D (n=15436)** | |  | **With T2D (n=3215)** | | P for interaction |
| --- | --- | --- | --- | --- | --- | --- |
|  | **OR 95% CI** | **P-value** |  | **OR 95% CI** | **P-value** |  |
| Blood urea nitrogen, (mmol/L) | 0.88 (0.84~0.92) | <0.001 |  | 0.99 (0.92~1.07) | 0.867 | 0.019 |
| Subgroups |  |  |  |  |  |  |
| Quartile 1 | 1.00(Ref) |  |  | 1.00(Ref) |  | 0.028 |
| Quartile 2 | 0.85 (0.74~0.98) | 0.030 |  | 0.74 (0.54~1.00) | 0.054 |  |
| Quartile 3 | 0.68 (0.58~0.79) | <0.001 |  | 0.99 (0.74~1.33) | 0.944 |  |
| Trend test |  | <0.001 |  |  | 0.937 |  |

*Adjusted for age, gender, BMI, race, educational level, smoking status, alcohol consumption, albumin, ALT, AST, creatinine, LDH, Uric acid, hypertension and* *physical activities.*
