# Supplementary material for: Associations between urea nitrogen and risk of depression among subjects with and without type 2 diabetes: A nationwide population-based study
Source: Front Endocrinol (Lausanne). 2022 Oct 27;13:985167. doi: 10.3389/fendo.2022.985167 (PMC9646599; doi:10.3389/fendo.2022.985167)
Supplement: Supplementary file 2 [file Table_2.docx]

**Supplementary Table S2.** Interactive effect of blood urea nitrogen and depression in patients with and without T2D (All participants). Blood urea nitrogen exclude（X±2SD）

| **Variable** | **Without T2D (n=15149)** | |  | **With T2D (n=3023)** | | P for interaction |
| --- | --- | --- | --- | --- | --- | --- |
|  | **OR 95% CI** | **P-value** |  | **OR 95% CI** | **P-value** |  |
| Blood urea nitrogen, (mmol/L) | 0.88 (0.83~0.92) | <0.001 |  | 1.00 (0.92~1.09) | 0.976 | 0.017 |
| Subgroups |  |  |  |  |  |  |
| Quartile 1 | 1.00(Ref) |  |  | 1.00(Ref) |  | 0.024 |
| Quartile 2 | 0.85 (0.73~0.98) | 0.025 |  | 0.73 (0.53~0.99) | 0.044 |  |
| Quartile 3 | 0.68 (0.58~0.80) | <0.001 |  | 0.96 (0.72~1.29) | 0.790 |  |
| Trend test |  | <0.001 |  |  | 0.893 |  |

*Adjusted for age, gender, BMI, race, educational level, smoking status, alcohol consumption, albumin, ALT, AST, creatinine, LDH, Uric acid, hypertension and* *physical activities.*
